# Characterization of Exercise-Induced Myocardium Growth Using Finite Element Modeling and Bayesian Optimization

**DOI:** 10.3389/fphys.2021.694940

**Published:** 2021-08-09

**Authors:** Yiling Fan, Jaume Coll-Font, Maaike van den Boomen, Joan H. Kim, Shi Chen, Robert Alan Eder, Ellen T. Roche, Christopher T. Nguyen

**Affiliations:** ^1^Cardiovascular Bioengineering and Imaging Laboratory, Cardiology Division, Massachusetts General Hospital, Charlestown, MA, United States; ^2^Department of Mechanical Engineering, Massachusetts Institute of Technology, Cambridge, MA, United States; ^3^Institute for Medical Engineering and Science, Massachusetts Institute of Technology, Cambridge, MA, United States; ^4^Athinoula A. Martinos Center for Biomedical Imaging, Charlestown, MA, United States; ^5^Harvard Medical School, Boston, MA, United States

**Keywords:** myocardium growth, exercise, finite element, Bayesian optimization, cardiac MRI

## Abstract

Cardiomyocyte growth can occur in both physiological (exercised-induced) and pathological (e.g., volume overload and pressure overload) conditions leading to left ventricular (LV) hypertrophy. Studies using animal models and histology have demonstrated the growth and remodeling process at the organ level and tissue–cellular level, respectively. However, the driving factors of growth and the mechanistic link between organ, tissue, and cellular growth remains poorly understood. Computational models have the potential to bridge this gap by using constitutive models that describe the growth and remodeling process of the myocardium coupled with finite element (FE) analysis to model the biomechanics of the heart at the organ level. Using subject-specific imaging data of the LV geometry at two different time points, an FE model can be created with the inverse method to characterize the growth parameters of each subject. In this study, we developed a framework that takes *in vivo* cardiac magnetic resonance (CMR) imaging data of exercised porcine model and uses FE and Bayesian optimization to characterize myocardium growth in the transverse and longitudinal directions. The efficacy of this framework was demonstrated by successfully predicting growth parameters of 18 synthetic LV targeted masks which were generated from three LV porcine geometries. The framework was further used to characterize growth parameters in 4 swine subjects that had been exercised. The study suggested that exercise-induced growth in swine is prone to longitudinal cardiomyocyte growth (58.0 ± 19.6% after 6 weeks and 79.3 ± 15.6% after 12 weeks) compared to transverse growth (4.0 ± 8.0% after 6 weeks and 7.8 ± 9.4% after 12 weeks). This framework can be used to characterize myocardial growth in different phenotypes of LV hypertrophy and can be incorporated with other growth constitutive models to study different hypothetical growth mechanisms.

## Introduction

is known to lead to chronic physiological changes in the cardiovascular system such as an increase in contractility and a decrease in vascular resistance, heart rate, and blood pressure as a results of parasympathetic mediation (Fernandes et al., 2011). In addition, it induces morphological changes to the heart, which are typically referred to as cardiac growth or hypertrophy. Cardiac growth can be categorized into two types at the macroscopic level: eccentric growth – where the ventricular volume increases, and concentric growth – where the ventricular wall thickness increases. At the microscopic level, growth is the result of increasing size of the cardiomyocytes and, similarly to the macroscopic observations, *in vitro* studies have shown that cardiomyocytes have two growth phenotypes: longitudinal and transverse sarcomerogenesis (Yang et al., 2016). Moreover, it has been hypothesized that longitudinal and transverse growth at the microscopic level, result in eccentric and concentric growth at the macroscopic level (Göktepe et al., 2010). These distinctions in growth types are important since different types of exercise produce different types of macroscopic growth – anaerobic exercise is typically associated with concentric growth, while aerobic exercise leads to eccentric growth (Mihl et al., 2008; Fernandes et al., 2011) – and, more importantly, growth can also be triggered by pathologic causes such as pressure-overload, with similar hypertrophic phenotypes, but leading to heart failure instead of improved cardiac function. The root cause of the discrepancy between physiologic and pathologic growth remains unclear except for histology studies showing that the latter is also accompanied by microstructure remodeling (e.g., interstitial fibrosis, non-uniform cardiomyocyte alignment, and excessive collagen deposition) (Vega et al., 2017).

There is a long history of studying myocardial growth experimentally, both *in vitro* and *in vivo* (Aboelkassem et al., 2019; Niestrawska et al., 2020). *In vitro* studies apply static loads on isolated cardiomyocytes in the longitudinal (Mansour et al., 2004) or transverse direction (Yang et al., 2016) to mimic the conditions of volume overload or pressure overload, respectively. These studies showed sarcomerogenesis in series or in parallel corroborates the current understanding of longitudinal or transverse growth in response to these pathological loadings. *In vivo* studies on cardiac growth rely on small and large animal models of pathological growth resulting from volume overload or pressure overload (Aboelkassem et al., 2019). Volume overload models, associated with eccentric hypertrophy, have been generated by either cutting the chordae tendineae to induce mitral regurgitation (Sahli Costabal et al., 2019; Li et al., 2020) or by implanting a pacemaker to repeatedly introduce premature ventricular contraction (PVC) (Torrado et al., 2021). Pressure overload models, which are usually linked to concentric hypertrophy, have been created by aortic banding (Olver et al., 2019; Torres et al., 2020), diet modification (Holzem et al., 2015; Olver et al., 2019), or genetic modification (LeGrice et al., 2012; Wilson et al., 2017). On the other hand, exercised-induced hypertrophic models have also been created in both small and large animals by swim training, wheel running, or treadmill running (Wang et al., 2010). Most of these *in vivo* studies evaluate the effects of growth on the cardiac function (e.g., ejection fraction, cardiac output, hemodynamics) as well as morphological changes of the left ventricular (LV) (e.g., relative wall thickness). A few studies have used histology, acquired either *ex vivo* at the end of the study or through invasive biopsy, to quantify the level of cardiomyocyte growth (Olver et al., 2019; Sahli Costabal et al., 2019; Li et al., 2020) or the changes in collagen fiber orientation (Torres et al., 2020). Due to the limitations associated with *ex vivo* analysis and the added complexity and risks of *in vivo* biopsies, there is a profound paucity of data on the microstructural changes of the myocardium during LV growth and remodeling. Thus, the mechanistic link of growth between the organ level and tissue–cellular level remains poorly understood.

Computational models that try to develop quantitative links between growth observations at the organ level and tissue–cellular level are promising tools to give better insights into growth mechanisms (Niestrawska et al., 2020). Currently, there are two main types of growth constitutive models: kinematic growth and constrained mixture growth. Kinematic growth is a phenomenon-based model which has been used to create finite element (FE) models for both concentric (Göktepe et al., 2010; Rausch et al., 2011; Genet et al., 2016) and eccentric hypertrophy (Göktepe et al., 2010; Genet et al., 2016; Sahli Costabal et al., 2019). Both stress-driven and strain-driven growth laws have been tested in these studies. Constrained mixture growth is a microstructure-based model. It has been used mostly in the context of vascular growth which involves simpler geometry and isotropic properties due to the associated complexity of implementation and high computational cost (Niestrawska et al., 2020).

Although computational models provide a powerful platform to test different hypothetical growth mechanisms, large amounts of experimental data either at the tissue level (for kinematic growth) or at the cellular level (for constrained mixture growth) are required to facilitate the simulations and validate the models. To date, histology is the most commonly used approach that can provide details about the microstructural changes of the myocardium. However, histology is typically limited to *in vitro* or *ex vivo* studies. Moreover, it is typically evaluated in a small number of regions with a reduced field of view. Consequently, it requires researchers to identify which areas are to be sampled beforehand and, more crucially, it is challenging to repeat longitudinally on the same subject without invasive biopsy. On the other hand, non-invasive imaging techniques such as cardiac magnetic resonance (CMR) can provide information about the macrostructural and functional changes of the heart in multiple pathological and physiological states, including cardiac remodeling (Anand et al., 2002; Sipola et al., 2011; Alkema et al., 2016). Moreover, the non-invasive nature of CMR allows imaging of the same subject at multiple time points, hence, enabling longitudinal studies. The main limitation of CMR compared to histology is its relatively low resolution, on the order of mm, which impedes the direct observation of cellular shape changes in the heart.^1^ In order to perform *in vivo* assessments of the microstructural changes occurring during diseases or exercise, it is necessary to bridge the gap between the macrostructural changes observed with CMR and the underlying microstructural changes in the myocardium.

With CMR data, FE can be used as a *forward* model to build subject-specific growth simulations and predict the LV morphological changes for given growth parameters. Assuming the governing laws of growth are valid, it is possible to estimate a set of growth parameters that predict the LV geometry observed post-growth from CMR using iterative optimization approaches. Such a technique would provide a quantitative link between growth in myocardial microstructure and morphological changes in the LV geometry. However, subject-specific FE models are computationally expensive and consequently running a large number of iterations within an optimization algorithm becomes prohibitive. In this context, Bayesian optimization (BO) was developed as a gradient-free optimization technique designed to optimize cost functions that are expensive to evaluate. Hence, BO can be used to optimize over parameterized FE models of the heart without evaluating a grid search, which could take weeks or months to compute per subject.

The aim of this work is to propose an optimization framework to estimate the microstructural changes in the myocardial tissue by combining CMR imaging with FE-based computational models and BO. In short, our approach parameterizes the possible myocardial growth mechanisms (e.g., transverse or longitudinal growth) within an FE model and then estimates the growth parameters that best describe the heart geometry observed with CMR after growth. Since the heart is imaged in its entirety and non-invasively, it is also possible to assess whole-heart changes and perform longitudinal studies to assess progression within the same subject. In this study, we illustrated the accuracy of the FE + BO framework by testing it on multiple synthetic and animal growth models. In all cases, initial and final (post-growth) geometries were obtained and the FE + BO algorithm was used to predict which combination of transverse/longitudinal microstructural growth occurred in the myocardium.

## Materials and Methods

**FIGURE 1:**
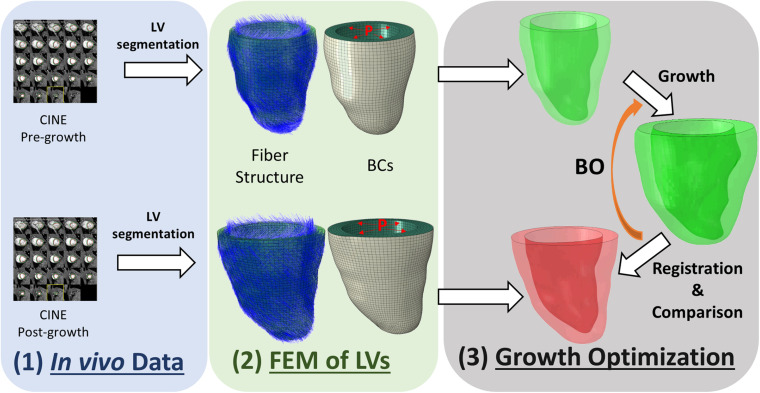
Overview of the workflow used to characterize cardiomyocyte growth. The workflow contains three modules: (1) *in vivo* data collection, (2) building finite element model (FEM) of the pre-growth and post-growth LV geometries with idealized fiber orientation and boundary conditions (BCs) such as end-diastolic pressure and pericardium constraint, and (3) growth optimization.

We developed an inverse-problem approach to non-invasively characterize cardiomyocyte growth from CMR and FE models, as described in Figure 1. Specifically, we acquired two CMR volumes of the LV at two time points – pre-growth (before starting exercise) and post-growth (after the exercise regime). Next, we built FE models of both LV geometries and applied hemodynamic loading and pericardial constraints to each. Finally, we applied cardiac growth to the pre-growth model and used it to estimate the microstructural cardiac growth parameters that best describes the macrostructural cardiac shape observed in the post-growth model. The overall method is composed of three main components, the myocyte growth model, the computational FE model and the estimation of the growth parameters performed with BO.

### Myocardium Growth Model

Since we used MRI data as the input information, kinematic growth was chosen instead of constrained mixture growth as the resolution of MRI is better suited for imaging at the macrostructural/tissue level. Kinematic growth theory introduces volumetric deformation to a continuum formulation with an approach similar to thermal-elastic coupling. In other words, the growth resulting from cardiomyocyte hypertrophy is modeled as volume increase in the myocardium. Under the kinematic growth framework, the total deformation gradient (**F**) can be multiplicatively decomposed into an elastic response (**F^*e*^**) and a growth multiplier (**F^*g*^**) as shown in Eq. 1. The former is used to determine the stress in the stress-strain constitutive model and the latter defines the growth magnitude in the three local orthogonal directions of the cardiac microstructure (fiber, sheetlet, and sheet-normal).

(1) $$\text{F}={\text{F}}^{e}\,{\text{F}}^{g}$$

As discussed earlier, cardiomyocyte has two main modes of growth, longitudinal and transverse growth, which correspond to series and parallel sarcomerogenesis, respectively. Therefore, we modeled the growth as transversely isotropic, where growth in the fiber direction is associated with longitudinal growth and growth in the sheetlet and sheet-normal directions are associated with transverse growth. The growth multiplier has the form:

(2) $${\text{F}}^{g}=\left(1+\alpha_{f}\right)\,\text{f}⊗\text{f}+\left(1+\alpha_{n}\right)\,\left(\text{n}⊗\text{n}+\text{s}⊗\text{s}\right),$$

where **f**, **s**, and **n** are unit vectors corresponding to the fiber, sheetlet, and sheet-normal directions that are orthogonal to each other. Similarly, α_*f*_ and α_*n*_ are the longitudinal and transverse growth coefficients.

For the elastic response of myocardium, the invariant-based hyperelastic model purposed by Holzapfel and Ogden (2009) was used. The strain energy density function of the model is shown in Eq. 3, where $I_{1}^{e}$, $I_{4f}^{e}$, $I_{4s}^{e}$, and $I_{8fs}^{e}$ are invariants of the right Cauchy green tensor (Ce=FeT⁢Fe) and *a*, *b*, *a*_*f*_, *b*_*f*_, *a*_*s*_, *b*_*s*_, *a*_*fs*_, and *b*_*fs*_ are material parameters (Holzapfel and Ogden, 2009). The “*a*” parameters have units of MPa and “*b*” parameters correspond to an exponential constant that is dimensionless. We adopted the material parameters characterized by Sack et al. (2018) from swine models, where *a* = 1.05 kPa, *b* = 7.542, *a*_*f*_ = 3.465 kPa, *b*_*f*_ = 14.472, *a*_*s*_ = 0.481 kPa, *b*_*s*_ = 12.548, *a*_*fs*_ = 0.283 kPa, and *b*_*fs*_ = 3.088.

(3) ψ=a2⁢b⁢exp⁢(b⁢(I1e-3))+∑i=f,sai2⁢bi⁢{exp⁢(bi⁢(I4⁢i-1e)2-1)}+afs2⁢bfs⁢[exp⁢(bfs⁢I8⁢f⁢se2)-1]

In the FE models which will be described in the next section, all the elastic material properties are kept constant while growth parameters α_*f*_ and α_*n*_ are varied from model to model in the workflow. With the kinematic growth frame work (Eq. 1), **F^*e*^** can be derived from **F** and **F^*g*^**, in which the former is computed as the gradient of the continuous deformation map and the latter is explicitly defined as in Eq. 2. The second Piola–Kirchhoff stress can then be computed from **F^*e*^** and strain energy density function (Eq. 3) as $\text{S}=\frac{\partial \psi }{\partial C^{e}}$. More details of the kinematic growth in the continuum mechanics framework are described in Genet et al. (2016).

### Finite Element Model

Finite element models (FEM) apply constitutive models that describe the growth behavior at the tissue level into each element and enable the evaluation of deformation and morphology changes at the organ level. To start building a FEM of the LV, a 3D volumetric model of its geometry is required. In this study, the LV geometries at end-diastole were generated from *in vivo* CMR imaging using semi-automatic segmentation tool Segment (Medviso) (Heiberg et al., 2010). In order to avoid through-slice discontinuities, the epicardium and endocardium contours from each slice were further smoothed by fitting a smoothing B-spline to the mask control points along the slice direction (Prakosa et al., 2014). The contours were used to create the 3D shape of the LV in FE software Abaqus 2018 (Dassault Systèmes, Providence, RI, United States) (Dassault Systèmes, 2018). The LV was meshed with hexahedron elements (C3D8) with element edge length of approximately 1.5 mm (i.e., a 1.5 mm × 1.5 mm × 1.5 mm element), resulting in 4–5 layers of elements across the myocardial wall. An idealized fiber orientation was applied using the Laplace–Dirichlet Rule-Based (LDRB) algorithm (Bayer et al., 2012) with epicardial–endocardial helix angle of −60° to 60°. Standard Abaqus user subroutines VUHYPER and VUEXPAN (Dassault Systèmes, 2018) were used to implement the Holzapfel–Ogden hyperelastic model and transversely isotropic growth model in Abaqus. To create pericardial constraints at the epicardium, a 3D shell geometry was obtained from the epicardial surface to model the geometry of the pericardium explicitly. The pericardium was meshed with quadrilateral shell elements (S4) and modeled as a linear elastic material with a Young’s modulus of 10 MPa (Lin et al., 2013). A frictionless contact interaction was applied between the epicardium surface (Γ_*epi*_) and the pericardium surface (Γ_*epri*_) using the penalty contact algorithm (Dassault Systèmes, 2018). A penalty pressure, which is linearly dependent on the overclosure distance (h), was applied on the two surfaces (Eqs 4a–c). A Dirichlet BC was applied at the basal plane (Γ_*base*_) and the basal ring of the pericardium (∁_*base*−*ring*_) to prevent movement of body in the longitudinal direction (Eq. 4d). A preload step followed by a growth step was implemented into the model. Assuming that the segmented LV geometry is closed to the stress-free configuration, an end-diastolic pressure (*p*_*ed*_) of 10 mmHg was applied on the endocardial surface to obtain the preloaded LV shape in the preload step. The LV pressure was kept constant in the growth step while kinematic growth in the transverse and longitudinal directions were implemented. All the BCs of the model are summarized in Eqs 4a–f.

(4a) $$\text{FSn}=p_{e\,p\,i}\,\text{n}\;o\,n\,\Gamma_{e\,p\,i}$$

(4b) $$\text{FSn}=-p_{e\,p\,i}\,\text{n}\;o\,n\,\Gamma_{p\,e\,r\,i}$$

(4c) $$p_{e\,p\,i}=2\,h$$

(where h is the overclosure distance between the two contacted surfaces)

(4d) $$u_{z}=0\;o\,n\;\Gamma_{b\,a\,s\,e},{∁}_{b\,a\,s\,e-r\,i\,n\,g}$$

(4e) $$\text{FSn}=p_{e\,n\,d\,o}\,\text{n}\;o\,n\;\Gamma_{e\,n\,d\,o}$$

(4f) $$p_{e\,n\,d\,o}=\left\{ \begin{array}{c} p_{e\,d}\,t\;t\in \left[0,1\right]\,\left(p\,r\,e\,l\,o\,a\,d\,s\,t\,e\,p\right)\\ p_{e\,d}\;t\in \left[1,2\right]\,\left(g\,r\,o\,w\,t\,h\,s\,t\,e\,p\right) \end{array}\right.$$

**FIGURE 2:**
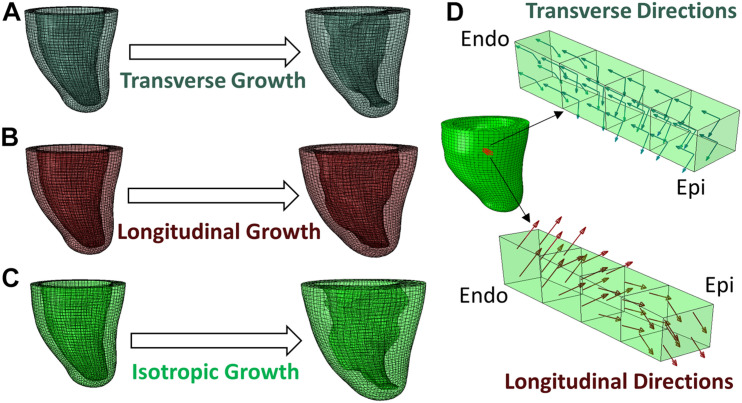
Different types of growth evolution predicted by the FE model, **(A)** transverse growth, **(B)** longitudinal growth, **(C)** isotropic growth. **(D)** Demonstration of transverse and longitudinal growth directions in a block of myocardium from endocardium to epicardium.

Due to non-linearities (large deformation, non-linear material model, and contact) in the model, the Abaqus/Explicit solver was used to conduct a quasi-static analysis (Dassault Systèmes, 2018). The Explicit Dynamic Analysis in Abaqus is designed to solve the dynamic equilibrium (Eq. 5). When the inertial force (**M**$\ddot{\text{u}}$) is small enough, the equation reduces to the static form of equilibrium and therefore leads to a quasi-static problem. The explicit solver uses the forward Euler method, in which the equations of motion are updated using previous information as shown in Eqs 6, 7. Preload and growth step time periods were set to 1 using a mass scaling technique and small stable time increments of 5 × 10^–6^ to ensure that the kinetic energy was negligible (<5%) compared to the total energy, as suggested in the Abaqus manual for quasi-static analysis (Dassault Systèmes, 2018). Hence, the “time” is an arbitrary value that indicates the loading magnitude but does not reflect the actual loading rate. For example, a growth simulation that linearly increases the transverse growth magnitude from 0 to α_0_ can provide intermediate outputs at time *t* ∈ [0, 1] as the solution of growth with transverse growth magnitude equals α_0_*t*. Figure 2 illustrates the macroscopic growth produced by three types of microscopic growth: transverse, longitudinal, and isotropic. Transverse growth increased the wall thickness of the LV, longitudinal growth dilated the LV chamber and isotropic growth resulted in both wall-thickening and LV chamber dilation. Both the transverse and longitudinal results agree with clinical observations and histological findings of eccentric and concentric hypertrophy (Gerdes, 2002).

(5) $$\text{M}\,\ddot{\text{u}}=\text{P}-\text{I}$$

(where **M** is the lumped element mass matrix, $\ddot{\text{u}}$ is acceleration, **P** is the external force vector, and *I* is the internal force vector)

(6) $${\text{u̇}}^{\left(i+\frac{1}{2}\right)}={\text{u̇}}^{\left(i-\frac{1}{2}\right)}+\frac{△\,t^{\left(i+1\right)}+△\,t^{\left(i\right)}}{2}\,{\text{ü}}^{\left(i\right)}$$

(7) $${\text{u}}^{\left(i+1\right)}={\text{u}}^{\left(i\right)}+△\,t^{\left(i+1\right)}\,{\text{u̇}}^{\left(i+\frac{1}{2}\right)}$$

(where **u** is displacement and $\dot{\text{u}}$ is velocity, the superscript (*i*) indicates the increment number and $i-\frac{1}{2}$ and $i+\frac{1}{2}$ refer to mid-increment values)

### Growth Parameter Estimation With Bayesian Optimization

The cardiac growth parameters (α_*f*_,α_*n*_) were estimated by maximizing the similarity between the LV geometries obtained from the growth FE model and from the LV geometry imaged with the second scan. Specifically, we maximized the DICE score (Dice, 1945) between the masks of the FE and CMR geometries (*M*_*FE*_(α_*f*_,α_*n*_)*andM*_*CMR*_, respectively):

(8) $$\max_{\alpha_{f},\alpha_{n}}\frac{2\,\left|M_{F\,E}\,\left(\alpha_{f},\alpha_{n}\right)\cap M_{C\,M\,R}\right|}{\left|M_{F\,E}\,\left(\alpha_{f},\alpha_{n}\right)\right|+\left|M_{C\,M\,R}\right|}$$

Unfortunately, the cost function in this maximization problem requires solving an FE growth model over the parameters (α_*f*_,α_*n*_) numerically. Hence, it is non-linear, does not have an analytical expression and each iteration is computationally expensive (around 2 h per iteration). These limitations impede using gradient-descent methods (Nocedal and Wright, 2000; Boyd and Vandenberghe, 2004) and is computationally infeasible for classical gradient-free methods (Nelder and Mead, 1965; Powell, 2009). Instead, we used BO, which is a gradient-free optimization method designed for problems whose cost function can only be evaluated at discrete points and which are expensive to compute (Jones et al., 1998; Osborne et al., 2009; Hutter et al., 2011). At each iteration, BO interpolates the cost function with a Gaussian process (Rasmussen and Williams, 2006) using the samples evaluated in previous iterations and then proposes a new point to evaluate within a bounded search space. The optimization is effectively performed in the process of proposing new points to evaluate. These are generated by maximizing an analytical acquisition function that balances the exploration of the search space against the exploitation of current local maxima to further improve the current best result. There have been multiple acquisition functions proposed in the literature, each providing different balances between exploration and exploitation (Kushner, 1964; Srinivas et al., 2010; Hoffman et al., 2011; Hernández-Lobato et al., 2015), and allowing for the introduction of non-linear constraints to the optimization (Hernández-Lobato et al., 2015; Ariafar et al., 2019). In this work, we used the Upper Confidence Bound (Srinivas et al., 2010), which maximizes the following trade-off between the mean μ(*x*) and variance σ(*x*) of the Gaussian Process, balanced by the scalar parameter β:

(9) $$\max_{x}\mu \,\left(x\right)+\beta \cdot \sigma \,\left(x\right)$$

As discussed, the cost function used in BO was the DICE score (DSC) between the masks of the predicted and imaged LV geometries. Evaluation of this cost function requires generating a 3D mask of the LV using the 3D coordinates of the nodes that constitute the FE mesh. In order to generate such mask, we determined which voxels in the 3D volume belong within the LV by interpolating a binary function in each voxel. Specifically, we used kernel density estimation with B-spline interpolation (kernel width of 4 voxels) and interpolated values of “1” at the position of the FE nodes. Finally, we implanted a threshold for the interpolated values at >0.25 and further filtered the resulting binary mask with a morphological closing filter with an element size of 6 voxels to avoid holes in the LV. To ensure that both the FE and CMR masks were aligned, we registered them with a rigid registration algorithm of their nodes in 3D (Myronenko and Song, 2009).

### Experiments

We tested our method with a series of synthetic experiments and further illustrated its application in a real scenario with animal models of exercise-induced cardiac growth. All experiments were done under IACUC-approved protocols at the Massachusetts General Hospital. Four Yucatan swine (2 months old) underwent treadmill exercise training for 12 weeks and were imaged *in vivo* at weeks 0, 6, and 12 after onset of exercise (one swine could not finish exercise before the submission). Cardiac imaging was performed on a 3T clinical MRI system (MAGNETOM Prisma or a Connectome, Siemens Healthineers, Erlangen, Germany) set at max 80 mT/m gradient strength and a standard 32-channel anterior–posterior surface coil. The animals were anesthetized, placed on a ventilator, and then imaged with a retrospectively ECG gated CINE MRI flow compensated gradient echo sequence (repetition time = 5.8 ms, echo time = 3.2 ms, flip angle = 20°, 4 averages, 1.4 mm × 1.4 mm × 2.5 mm, 25 cardiac phases).

After imaging, the LV at end-diastole was segmented to generate an FE model as described in the previous section. The FE model and the LV masks at weeks 6 and 12 were then introduced into the optimization framework to estimate the transverse (α_*n*_) and longitudinal (α_*f*_) growth of the myocardium. The optimization was performed in python using the BO implementation in the BoTorch package (Balandat et al., 2020) with UCB as the acquisition function. The parameter β, which balances exploration and exploitation in UCB, was somewhat arbitrarily set to 10 since it provided balance between the mean and variance of the Gaussian Process estimate after initialization. The maximum growth was set to 1 (equivalent to doubling of size), resulting into a search space bounded between 0 and 1 for both growth parameters. The optimization was initialized with 3 samples of growth parameters set to [0, 1], [1, 0], and [1, 1] and BO was run for 10 iterations. Given the numerical nature of the quasi-static FE model, it provided intermediate outputs of growth that could be used as additional samples within the Gaussian Process fitting in BO. Consequently, each growth simulation provided five valid cost-function evaluations between zero-growth and the selected combination of transverse and longitudinal growth parameters, and these were introduced into each iteration of the BO algorithm to improve the estimate of the Gaussian Process.

In order to evaluate the results, synthetic growth was applied to three LV geometries from the previously described swine models. For each LV geometry, the ventricle was modified with six randomly prescribed transverse and longitudinal growth parameters. The growth parameters were set to be equal across geometries to reliably compare the results across subjects. Hence, the resulting synthetic dataset consisted of a total of 18 simulations (3 geometries × 6 growth realizations), each with known ground truth for their respective growth parameters. In order to avoid committing an *inverse crime* “noise” was added in the form of forward model differences between the generation of the synthetic data and the model used within the optimization. Specifically, the synthetic data were generated with increased spatial resolution in the FE meshes (element size reduced to 1 mm from 1.5 mm) and smaller increment step size in the quasi-static growth model (reduced from 5 × 10^–6^ to 1 × 10^–6^) in the Abaqus/Explicit solver.

We evaluated the parameter estimation error in the synthetic experiments with the normalized prediction error between the ground truth $\left(\alpha_{f}^{G\,T},\alpha_{s}^{G\,T}\right)$ and predicted $\left(\alpha_{s}^{p},\alpha_{f}^{p}\right)$ growth parameters:

(10) E⁢r⁢r⁢o⁢r=(αfG⁢T-αfp)2+(αsG⁢T-αsp)2αfG⁢T2+αsG⁢T2

For all experiments, including the real-case examples, we report the final DICE score (DSC) between the predicted growth model and the true LV geometry, and illustrate the similarity between LV geometries with 3D plots, as well as contour plots of the LV masks in short and long axis views.

## Results

**FIGURE 3:**
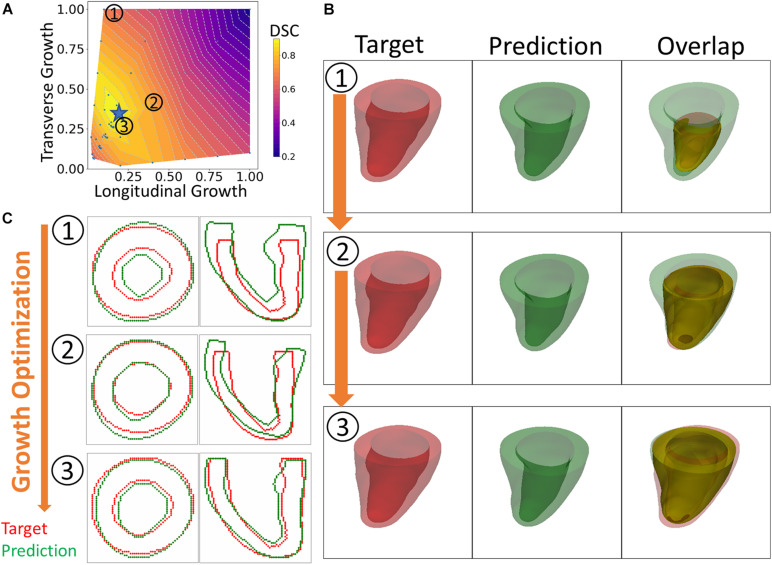
An overview of the results. **(A)** Heatmap of DICE scores (DSC) for different sets of growth parameters. **(B)** 3D views comparing predicted and targeted geometries at three different scenarios indicated on the heatmap. **(C)** 2D contour comparison of predicted and targeted geometries from the long and short axis views. (1) to (3) indicate three different sets of growth parameters that were tested during the optimization process. From (1) to (3), the BO method increasingly finds solutions that improve the DICE score.

An overview of the outputs generated with the FE + BO framework is shown in Figure 3. The heatmap in Figure 3A shows the DICE score distribution across two axes of transverse and longitudinal growth parameters. Regions with high DICE score were indicative of good alignment between the predicted and true geometries and the parameters with the highest DICE score (typically >90%) were identified as the final prediction (indicated with a blue star). The LV geometries that correspond to several iterations in the BO optimization are shown in Figures 3B,C. The 3D views (B) provide clear morphology of the predicted and imaged LVs and the yellow intersection illustrates the volume overlap between them after registration. Similarly, the 2D contours (C), provide a more detailed comparison between target and prediction in two planes. Figure 3 illustrates three samples obtained along the optimization and sorted from low to high DICE scores.^2^ The first example (top row) with parameters α_*f*_ = 0.1 and α_*n*_ = 1 showed a thickened LV wall and an elongated chamber, compared to the target LV. The second example (middle row) presented lower transverse growth but higher longitudinal growth (α_*f*_ = 0.4 and α_*n*_ = 0.4). In this case, the geometry was more similar to the target one and was characterized by smaller wall thickness and smaller apex-to-base distance. Due to larger longitudinal growth, the chamber was more dilated in the radial direction, compared to the targeted LV. The best example (bottom row) was found for parameters α_*f*_ = 0.17 and α_*n*_ = 0.33. Both the 2D contours and the 3D plots show improved similarity with the target LV than that obtained with the previous examples, albeit the LV size was slightly under-predicted.

**FIGURE 4:**
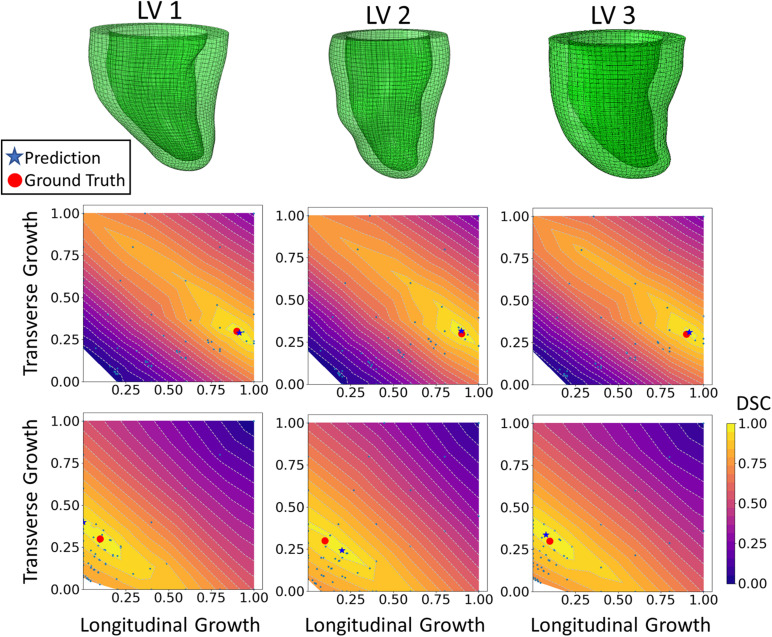
DICE score heatmap results of the synthetic experiments. The top row shows the three LV geometries that were used in the synthetic experiments. The final prediction and ground truth are indicated in each heatmap as a blue star and red dot, respectively. For each LV geometry, two out of all six cases are shown. The middle row includes examples of growth that is largely dominated by longitudinal growth (α_*f*_ = 0.9, α_*n*_ = 0.3) and the bottom shows examples of growth that is largely dominated by transverse growth (α_*f*_ = 0.1, α_*n*_ = 0.3).

**FIGURE 5:**
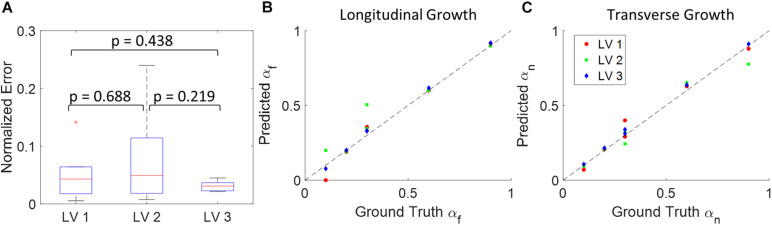
Quantitative analysis of the results from 18 synthetic experiments. **(A)** Boxplot of normalized error across different subjects. (Boxplots show median, interquartile ranges, and whiskers show range. *P*-values were calculated using standard *t*-test). **(B)** Scatter plot of predicted longitudinal growth vs. ground truth longitudinal growth. **(C)** Scatter plot of predicted transverse growth vs. ground truth longitudinal growth. Dash lines in panels **(B,C)** indicate the identity line where predictions with zero error should locate on.

**FIGURE 6:**
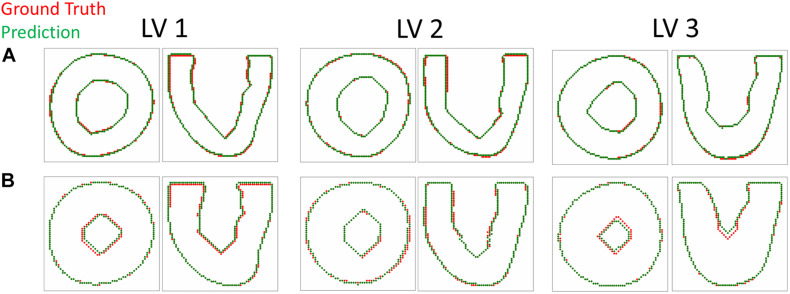
Contour plots comparing the predicted and target LV geometry from the short axis and long axis views. The top row **(A)** includes the longitudinal growth examples (α_*f*_ = 0.9, α_*n*_ = 0.3) and the bottom row **(B)** includes the transverse growth examples (α_*f*_ = 0.1, α_*n*_ = 0.3).

The DICE score heatmaps of the synthetic experiments are shown in Figure 4. These illustrate how the FE + BO framework was capable of estimating growth parameters in the synthetic models. In all cases, the DICE score heatmaps resulted in a single local maximum with a peak in the vicinity of the true parameters. Consequently, the estimated growth parameters were similar to those of the ground truths across different LV geometries and growth scenarios. Figures 5B,C shows a scatter plot with the estimated and true growth parameters. Both the estimated transverse and longitudinal growth resulted in good alignment with the ground truth (points are near the identity line), although these were, respectively, underestimated and overestimated (below and above the identity line). Quantitatively, the normalized error of the growth parameters, shown in Figure 5A, was 5.5 ± 5.8% and there was no significant difference in error across different LV geometries. The 2D contours of the predicted and true masks are shown in Figure 6. These resulted in good subjective alignment of the predicted LV geometry and that of the ground truth.

**FIGURE 7:**
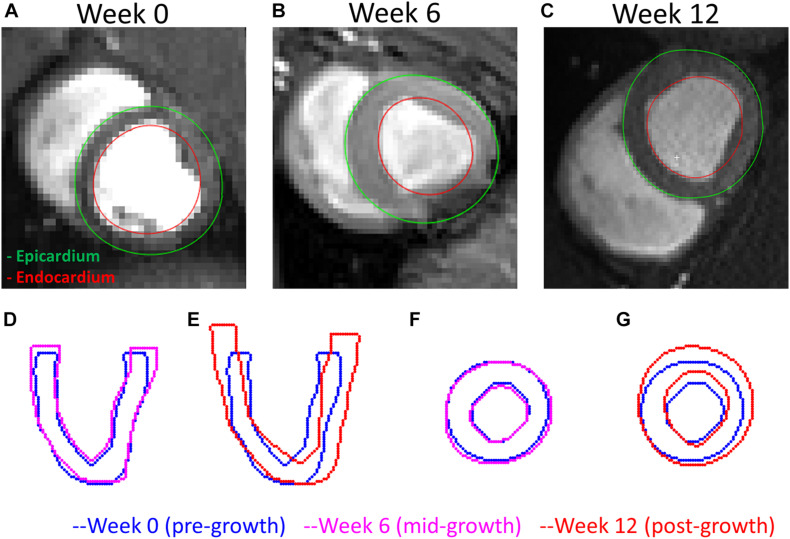
A comparison of the LV geometries before and after exercise-induced growth. **(A–C)** MRI short-axis views of the LVs at weeks 0, 6, and 12 during exercise training. **(D,E)** Long-axis views comparing weeks 0–6 and 12 LV geometries. **(F,G)** Short-axis views comparing weeks 0–12 LV geometries. Rigid registrations were performed between the two geometries in panels **(D–G)**.

**FIGURE 8:**
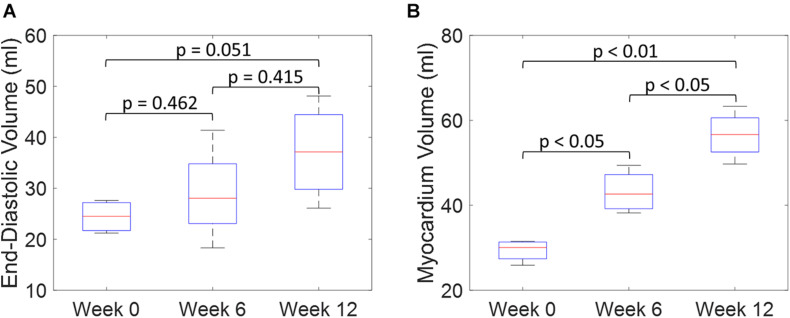
Quantitative comparisons of LV end-diastolic volume **(A)** and myocardium volume **(B)** between weeks 0, 6, and 12 during exercise training. (Boxplots show median, interquartile ranges, and whiskers show range. *P*-values were calculated using standard *t*-test).

An example of the true end-diastolic LV geometries segmented from MRI along different time points during the exercise training (weeks 0, 6, and 12) is shown in Figure 7. Figures 7A–C show examples of the epicardial and endocardial contours identified from short-axis CINE slices under different training time points. From Figures 7D,E, the long-axis view comparisons after rigid registration between the two geometries show LV chamber elongation and dilation is relatively minimum at week 6 but substantial at week 12. Similarly, the short-axis views (Figures 7F,G) show that wall thickening effect is more evident in week 12 than week 6. Quantitative evaluation of LV shape changes during exercise training are shown in Figure 8. The LV (*n* = 4) shows an increased end-diastolic (ED) volume (Figure 8A), and a significant increase in myocardial volume (Figure 8B) as the exercise program progresses. These results are consistent with eccentric hypertrophy.

**FIGURE 9:**
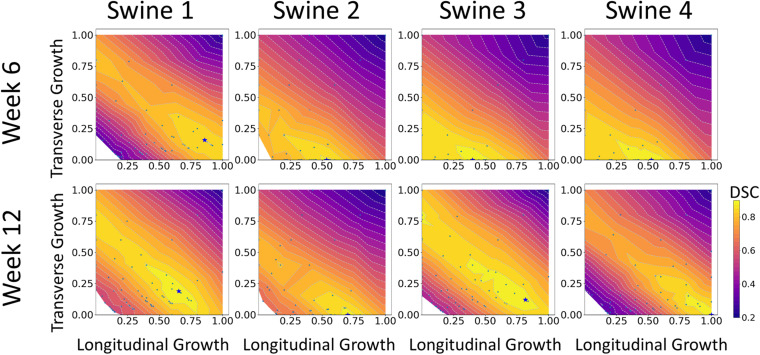
DICE score heatmaps of growth parameter prediction for four exercised animals at two different time points (weeks 6 and 12).

The results of growth characterization on these four exercised animals are shown in Figure 9. As reported in the synthetic experiments, all DICE score heatmaps resulted in a single local maximum within the search space. Since this data was obtained *in vivo*, there is no ground truth for the growth parameters. However, the estimated parameters consistently resulted in larger longitudinal growth than transverse growth. In fact, transverse growth values were almost negligible for most of the cases while a continuous increase in longitudinal growth was observed between weeks 6 and 12, except for Swine 1. On average, all animals (*n* = 4) that underwent exercise training resulted in growth parameters (α_*f*_ = 0.580 ± 0.196 and α_*n*_ = 0.040 ± 0.080) at week 6 and (α_*f*_ = 0.793 ± 0.156 and α_*n*_ = 0.078 ± 0.094) at week 12. The estimated growth parameters for each swine and session are reported in Table 1.

**TABLE 1:** Predictions of growth parameters in four exercised animals at two time points (weeks 6 and 12).

	**Swine 1**		**Swine 2**		**Swine 3**		**Swine 4**	
	α_**f**_	α_**n**_	α_**f**_	α_**n**_	α_**f**_	α_**n**_	α_**f**_	α_**n**_
Week 6	0.86	0.16	0.54	0.00	0.40	0.00	0.52	0.00
Week 12	0.65	0.19	0.70	0.00	0.82	0.12	1.00	0.00

**FIGURE 10:**
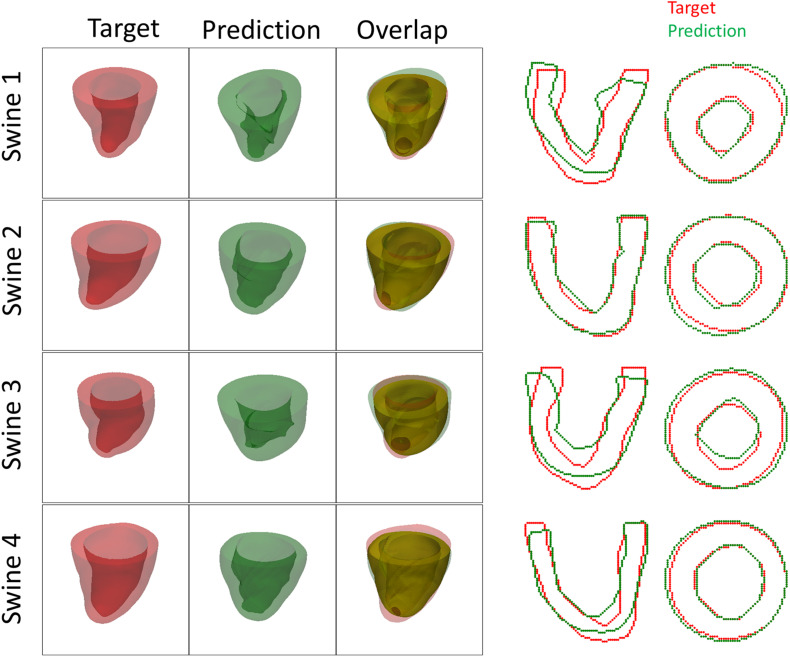
Plots of 3D geometries and 2D LV contours to compare the predicted and targeted LV geometries of exercised animals at week 12.

**FIGURE 11:**
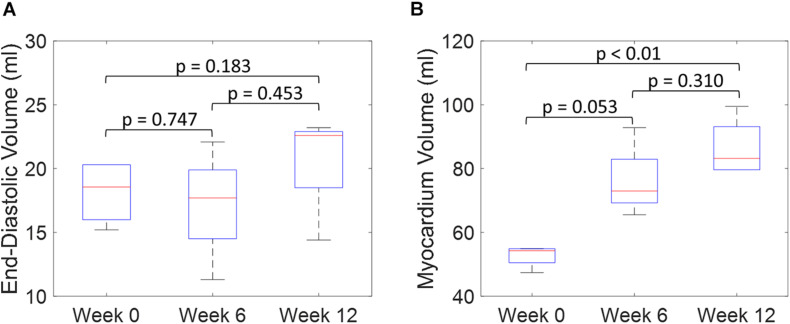
Quantitative comparison of end-diastolic volume **(A)** and myocardium volume **(B)** between the preloaded LVs at week 0 and the predicted growth LVs at weeks 6 and 12. (Boxplots show median, interquartile ranges, and whiskers show range. *P*-values were calculated using standard *t*-test).

Both the 3D plots and 2D contours of the predicted and target LV geometries are compared in Figure 10 for all four animals at week 12. Both visualizations of the LV geometries show that the FE + BO framework was able to find growth parameters that resulted in similar predicted LV geometries to those observed in the *in vivo* data. The 2D long-axis views show that the method tends to underpredict chamber elongation, except for Swine 2. On the contrary, overprediction on wall thickening is shown in the short-axis views. From the 3D overlapping views, it is clear that rigid registration realigned the two geometries before calculating the DSC. ED volume and myocardial volume of the preloaded LVs and growth model predicted LVs at weeks 6 and 12 are shown in Figure 11. The optimized growth simulations predicted a continuous increase of myocardium volume at weeks 6 and 12 similar to experimental measurements in Figure 8B. However, the trend for ED volume elevation, which is shown in the experimental data, was not reproduced in the growth simulations. This indicates that the pericardium constraint may have been over-estimated in the FE model such that longitudinal growth did not provide a sufficient level of LV chamber dilation. Overall, the method shows that exercise growth is more prone to longitudinal growth than transverse growth.

## Discussion

The results of synthetic experiments suggest that the proposed FE + BO framework is capable of estimating the growth parameters of the myocardium with inputs of pre- and post-growth LV geometries. The overall normalized error was 5.5 ± 5.8% and there were no significant differences across heart geometries. In the cases with lower level of growth, especially in the longitudinal direction, the predictions have higher errors. This indicates that the DSC score is more sensitive to transverse growth than longitudinal growth. All DICE scores at the optimal parameters were higher than 90%, presented a single global maxima and the optimized LV geometries were similar to their corresponding ground truths (Figure 6), providing confidence on the stability of the estimated parameters.

Moreover, the results from the animal model predicted significantly higher levels of longitudinal growth (58% for week 6 and 79.3% for week 12) than transverse growth (4% for week 6 and 7.8% for week 12). Longitudinally, all animals show an increase of growth level in the longitudinal direction from weeks 6 to 12, expect for Swine 1 in which the predicted level of longitudinal growth reduces from 86% at week 6 to 65% at week 12. Although minimal, the transverse growth level predicted in Swine 1 and 3 also increases over time during the exercise training process. Performing such longitudinal analysis without the FE + BO framework would only be possible with invasive and potentially hazardous biopsies of the heart. Overall, the growth characterization results suggest that exercise-induced myocardial growth is more prone to longitudinal growth. This is not only consistent with the qualitative LV imaging comparisons showing LV elongation and dilation (Figures 7, 8), but also agrees with the literature where running – categorized as aerobic exercise – has been reported to lead to eccentric hypertrophy with longitudinal growth at the cardiomyocyte level in different species (Mihl et al., 2008; Fernandes et al., 2011). However, the predicted level of growth in the longitudinal direction is much higher than reported cardiomyocyte dimensional increase (15–35%) from literature (Wang et al., 2010). This discrepancy is likely due to the over-simplified FE model with generalized material properties, fiber orientation and hemodynamic BCs such that it cannot simultaneously represent concentric and eccentric hypertrophy. In order to refine the subject-specific model, myocardium properties can be characterized using CINE data and dynamic LV models and more realistic fiber orientation can be assigned using cardiac structural information from diffusion tensor imaging data (Sack et al., 2016). While LV pressure is difficult to assess non-invasively, the preload step could be improved by using the early-diastolic filling geometry (Zhang et al., 2018) instead of the end-diastolic geometry as the reference configuration such that the preloaded LV configuration is more representative of the ED state. Despite its computational cost, an even more rigorous approach would be to use inverse methods to identify the stress-free LV configuration so that the subsequent preloaded LV geometry should be equivalent to the true ED geometry (Rausch et al., 2017; Wang et al., 2020). Moreover, the cardiac growth process in the swine models was monitored from 2 to 6 months old during which time the animals also grow in size. Thus, the results we are seeing may not only contain exercise-induced growth but also physical growth where the LV mass increases as the body weight increases. Further validation of our results with histology is warranted.

The current growth model was designed to characterize growth with two unique parameters for the entire geometry. However, spatially heterogeneous growth is prevalent in patients with hypertrophic cardiomyopathy (Maron et al., 2009). To address spatially dependent characterizations, the current model could be extended to include a parameterized spatial distribution of growth and optimize those parameters. Moreover, this framework can be further extended to more sophisticated growth laws (e.g., stretch-driven growth and strain-driven growth). An example of such a model is the work by Sahli Costabal et al. (2019), who introduced a probabilistic model to connect sub-cellular remodeling to strain-driven myocardium growth. Combination of this method with our current FE + BO framework and optimization of biologically significant parameters such as magnitude, rate, and biomechanical driving factors of growth could yield interesting mechanistic findings. To further improve the capability of our framework to investigate growth at the cellular level, a constrained mixture model can be incorporated. Despite its complexity and high computational cost, this model can provide a more powerful framework to reveal the mechanistic link between biomechanics at the organ level and biological factors at the tissue–cellular level (Niestrawska et al., 2020). Implementing these growth models into our framework would enable efficient *in silico* testing of different growth hypotheses with multi-scale models.

Furthermore, this framework is not limited to growth parameter characterization. Ideally, it can be used as a generic method to characterize material parameters as long as the undeformed and deformed geometries of the object are given in the application. Theoretically, it would be possible to run a grid search parametric study to determine the optimal parameters in these models. However, grid search quickly becomes computationally intractable in the context of FE models due to their expensive computationally costs (around 2 h with 10 CPUs for each evaluation). For example, for an accuracy of 90% in the growth model presented, it would be needed to compute a grid search with spacing of 0.05. This search would require computing 400 simulations, resulting in 800 h (33.3 days) of computation. Instead, the FE + BO approach resolved the maxima within 10 iterations, corresponding to about 20 h of computation. Similarly, classical optimization methods (e.g., Simplex, Monte-Carlo) would not be feasible due to the high computational costs of each FE model evaluation. These limitations are set to increase with more complex growth models (longer compute time) or increased dimension of the parameterization (exponentially larger search space). Moreover, the current FE + BO method could be further modified to improve its accuracy and speed-to-convergence. One immediate source of improvement is to modify the acquisition function to incorporate knowledge of the multiple samples generated during the quasi-static FE model evaluations. Currently, we incorporate these samples in the Gaussian process estimation, but the optimization of the acquisition function is done with off-the-shelf UCB, which assumes a single evaluation of the cost function will be provided. This modification would facilitate more efficient sampling of the search space in each BO iteration. Similarly, the selection of the trade-off β parameter in UCB should be done more systematically before the first iteration to balance the mean and variance of the Gaussian Process estimated during initialization.

### Limitations

The experimental limitations arise from two aspects: (1) acquisition of the MRI data and (2) segmentation of the LV geometry. The MRI data was acquired with two different scanners with different resolutions (mostly 1.4 × 1.4 × 2.5 mm with two exceptions of 1.3 × 1.3 × 2.5 mm and 1.8 × 1.8 × 6 mm). Lower resolution could reduce the accuracy of segmented LV geometry. The data was acquired along the short axis of the LV, and the actual positions of where the first and last slices reach the base and apex of the LV affect the length of the reconstructed LV geometry. Slice thickness of 2.5 mm is large enough to compare the growth magnitude, especially in the longitudinal direction. Therefore, one or two long axis views of MRI should be acquired and used in future segmentations. A semi-automatic segmentation approach was used in this study and then manually corrected to identify the LV contours in Segment (Medviso) (Heiberg et al., 2010). Further, there are motion artifacts and distortion around the free wall due to field homogeneity caused by the liver. The automatic segmentation method underperforms in these regions and manual corrections are subjective. A more robust automatic segmentation method should be used with minimal manual correction in order to increase reproducibility and reduce human bias.

Another limitation of this framework is introduced by the selection of BCs and tissue properties in the FE model. Model mis-specification can lead to errors in the optimization and result in unrealistic growth parameters. Identifying which models and parameters are most important for an accurate growth selection will be essential in future work. During the development of this study, we found that pericardial constraints are critical for creating realistic concentric hypertrophy in the transverse growth model. In this context, constraints from the pericardium and surrounding tissue at the epicardium surface is even more difficult since there is no clear consensus in the literature about what model to use for dynamic heart modeling. Some studies propose explicitly creating surrounding structures (Fritz et al., 2014), while others propose using “spring-dashpot” surrogates (Pfaller et al., 2019) to apply the constraints in dynamic heart modeling. Both models demonstrated the importance of including pericardial constraints on dynamic heart modeling. However, these models might not be applicable in the context of cardiac growth modeling since the heart undergoes gradual deformation at a much longer time scale. Within these time periods, the pericardium and surrounding tissue are likely to undergo their own remodeling, hence changing the constraints to the LV growth. Applying a constant linear elastic material models on the pericardium is likely to over-constrain the epicardium resulting in severe wall-thickening and chamber volume reduction which is shown in Figure 11. For future work, it will be important to consider the remodeling of pericardium and surrounding tissue so that more realistic BCs can be applied to the FE model. In addition to the pericardial constraints, sensitivity studies on the LV pressure BC and the initial configuration should be conducted. In this study, the ED state was used for the initial configuration since it is the state that can be consistently identified with CINE MRI and is a geometry that is relatively unaffected by external forces compared to the end-systolic state. For future studies, the growth simulation could be initiated from alternative configurations in the diastolic part of the cardiac cycle (e.g., early-diastolic filling, or diastasis) with different diastolic pressure BCs to check whether the growth optimization results are sensitive to any of these variations.

A limitation of the synthetic experiments is the simplistic source of “noise” added to the generated data which could lead to an overestimation of the accuracy of the synthetic results. In future work more representative noise could include segmentation variability (Tate et al., 2020), and the post-growth geometry could be generated with a more biologically relevant growth model (e.g., stress/strain-driven growth or constrained mixture growth) to further evaluate the framework performance. However, with our current implementation, such growth models are computationally impractical for whole LV geometries. Further validation is warranted for the animal experiments by comparing histological imaging results to the growth parameters estimated by the model (Sahli Costabal et al., 2019).

## Conclusion

In summary, this study introduces a Bayesian optimized framework that can be used to non-invasively characterize growth at the tissue level at multiple time points. The FE modeling in this framework enables discernment of mechanistic links between macrostructural imaging and microstructural changes at the tissue level. As such, we believe that the framework can be a powerful tool to reveal fundamental insights into myocardial growth and remodeling mechanisms. In the future, this framework could facilitate the longitudinal study of multiple physiological and pathological conditions and may have practical utility in assessing cardiac disease progression or response to therapy.

## Data Availability Statement

The original contributions presented in the study are included in the article/supplementary material, further inquiries can be directed to the corresponding author/s.

## Ethics Statement

The animal study was reviewed and approved by the Institutional Animal Care and Use Committee (IACUC) at Massachusetts General Hospital.

## Author Contributions

YF, JCF, ETR, and CTN designed the study, analysis the data, and wrote the manuscript. JCF, MvdB, JHK, SC, and RAE conducted the *in vivo* experiments. YF and JCF conducted the *in silico* experiments. All authors contributed to the article and approved the submitted version.

## Conflict of Interest

The authors declare that the research was conducted in the absence of any commercial or financial relationships that could be construed as a potential conflict of interest.

## Publisher’s Note

All claims expressed in this article are solely those of the authors and do not necessarily represent those of their affiliated organizations, or those of the publisher, the editors and the reviewers. Any product that may be evaluated in this article, or claim that may be made by its manufacturer, is not guaranteed or endorsed by the publisher.

## Footnotes

1 There exist MRI-based technologies, such as T1, T2, or diffusion CMR, which can provide microstructural information of the tissue, but not direct observations of the shape of the myocytes.

2 Note that, given the exploration–exploitation nature of BO, there is no guarantee that the scores obtained along consecutive iterations are monotonically increasing.
